# Anemia and cerebrovascular disease: pathophysiological insights and clinical implications

**DOI:** 10.1097/MS9.0000000000002907

**Published:** 2025-05-21

**Authors:** Emmanuel Ifeanyi Obeagu, Getrude Uzoma Obeagu

**Affiliations:** a Department of Biomedical and Laboratory Science, Africa University, Mutare, Zimbabwe; b School of Nursing Science, Kampala International University, Kampala, Uganda

**Keywords:** anemia, cerebrovascular disease, hemoglobin, pathophysiology, stroke

## Abstract

Anemia and cerebrovascular disease (CVD) are interconnected conditions that significantly impact global health, often coexisting to worsen clinical outcomes. Anemia-induced hypoxia increases the production of reactive oxygen species (ROS) via mitochondrial dysfunction and xanthine oxidase activity, amplifying oxidative stress. ROS directly harm endothelial cells by disrupting tight junctions, increasing vascular permeability, and compromising the integrity of the blood-brain barrier. This review delves into the pathophysiology of anemia in the context of CVD, emphasizing the impact of hypoxia, oxidative stress, and endothelial dysfunction. Clinically, anemia worsens CVD prognosis by elevating the risk of ischemic and hemorrhagic strokes, delaying recovery, and increasing mortality rates. If untreated, anemia exacerbates neuronal damage, promotes vascular remodeling, and perpetuates systemic inflammation, leading to poor functional outcomes. This review underscores the necessity of timely anemia diagnosis and targeted management in patients with cerebrovascular disease to mitigate these adverse outcomes.

## Introduction

Anemia, characterized by a deficiency in the number of red blood cells or hemoglobin concentration, is a prevalent global health issue with significant implications for cerebrovascular health. The interplay between anemia and cerebrovascular complications has garnered increasing attention in medical research and clinical practice due to its profound impact on neurological function and overall well-being. Understanding the intricate relationship between anemia and cerebrovascular disease is essential for effective diagnosis, management, and prevention strategies^[1]^. Cerebrovascular diseases encompass a spectrum of conditions affecting the blood vessels supplying the brain, including ischemic stroke, hemorrhagic stroke, and vascular dementia. These conditions are major contributors to morbidity and mortality worldwide, leading to significant disability and healthcare costs. Anemia, by compromising the oxygen-carrying capacity of blood, exacerbates the pathophysiological mechanisms underlying cerebrovascular diseases, thereby increasing the risk of adverse outcomes^[2]^. The effects of anemia on cerebrovascular health are multifaceted and extend beyond simple oxygen deprivation. Anemia-induced hypoxia triggers a cascade of physiological responses, including oxidative stress, inflammation, endothelial dysfunction, and impaired neurovascular coupling. These processes disrupt the delicate balance between oxygen supply and demand in the brain, leading to neuronal injury, cognitive impairment, and an increased susceptibility to cerebrovascular events^[3]^. Oxidative stress, arising from an imbalance between reactive oxygen species (ROS) production and antioxidant defenses, is a central mechanism linking anemia to cerebrovascular complications. ROS, generated during hypoxia and ischemia, damage cellular components and disrupt critical signaling pathways involved in vascular homeostasis. This oxidative damage contributes to endothelial dysfunction, blood-brain barrier disruption, and neuronal injury, amplifying the detrimental effects of anemia on cerebrovascular health^[4]^.

Inflammation further exacerbates the pathophysiology of anemia-related cerebrovascular complications. Anemia-induced hypoxia and oxidative stress stimulate the production of pro-inflammatory cytokines and chemokines, activating immune cells and perpetuating a chronic inflammatory state. This inflammatory cascade disrupts endothelial function, promotes atherosclerosis, and impairs neurovascular coupling, leading to increased susceptibility to stroke and cognitive decline^[1]^. Endothelial dysfunction, characterized by impaired vasodilation, increased vascular permeability, and pro-thrombotic states, is a hallmark of anemia-related cerebrovascular disease. Anemia-induced hypoxia and oxidative stress compromise endothelial integrity, disrupting the dynamic regulation of cerebral blood flow. This endothelial dysfunction contributes to microvascular pathology, blood-brain barrier disruption, and neuroinflammation, exacerbating the risk of cerebrovascular events^[2]^. Neurovascular coupling, the tight coupling between neuronal activity and cerebral blood flow, is crucial for maintaining brain function and metabolic homeostasis. Anemia disrupts this intricate relationship through various mechanisms, including reduced oxygen supply, impaired nitric oxide production, astrocyte dysfunction, and inflammatory responses. These disruptions compromise the brain’s ability to regulate blood flow in response to neuronal activity, contributing to cognitive impairment and increasing the risk of cerebrovascular events^[3]^.

Cerebrovascular disease (CVD) are two significant public health concerns with substantial global prevalence and impact. Anemia, affecting over 1.6 billion individuals worldwide, is a leading cause of disability and accounts for a considerable burden in low- and middle-income countries (LMICs). The prevalence of anemia is particularly high among vulnerable populations such as women, children, and the elderly, with iron deficiency contributing to 50% of cases. Concurrently, cerebrovascular disease, the second leading cause of death globally, affects approximately 15 million people each year, resulting in 5 million deaths and 5 million cases of permanent disability. The coexistence of these conditions has profound implications for patient outcomes, highlighting the need for a deeper understanding of their interplay. The pathophysiological link between anemia and cerebrovascular disease lies in several interconnected mechanisms, including oxidative stress, inflammation, endothelial dysfunction, and impaired neurovascular coupling. These processes create a vicious cycle that exacerbates neurological damage and impairs recovery after cerebrovascular events such as stroke^[3,4]^.

## Aim

The aim of this review is to comprehensively examine the impact of anemia on cerebrovascular health, elucidating the underlying pathophysiological mechanisms and exploring the clinical implications.

## Rationale

The rationale for conducting this review stems from the growing recognition of the intricate interplay between anemia and cerebrovascular disease and its significant clinical implications. While anemia has long been recognized as a risk factor for cardiovascular disease, its impact on cerebrovascular health has garnered increasing attention in recent years. Cerebrovascular diseases, including stroke and vascular dementia, are leading causes of morbidity and mortality worldwide, and anemia exacerbates the pathophysiological processes underlying these conditions. Anemia-induced hypoxia and oxidative stress have profound effects on cerebral blood flow regulation, endothelial function, and neurovascular coupling, thereby increasing the risk of cerebrovascular events. Secondly, individuals with anemia may present with cognitive impairment, which can be exacerbated by cerebrovascular disease. Thirdly, optimizing the management of anemia in patients with cerebrovascular disease may improve outcomes and reduce the burden of disability. By elucidating the complex interactions between anemia and cerebrovascular disease, this review aims to provide insights into potential therapeutic targets and management strategies. By addressing both the underlying causes of anemia and its cerebrovascular consequences, clinicians can develop more effective approaches to prevent, diagnose, and treat cerebrovascular diseases in individuals with anemia, ultimately improving patient outcomes and enhancing quality of life.

The interplay between anemia and CVD represents a critical area of investigation due to its profound impact on clinical outcomes and public health. Anemia, characterized by reduced hemoglobin levels, impairs the oxygen-carrying capacity of the blood, leading to tissue hypoxia. The brain, being highly sensitive to oxygen deprivation, is particularly vulnerable, and the resulting hypoxia can exacerbate the pathophysiology of cerebrovascular disease^[5]^. Anemia disrupts oxygen delivery to tissues by reducing the hemoglobin concentration and, consequently, the blood’s oxygen-carrying capacity. In response to hypoxia, mitochondrial respiration becomes inefficient, resulting in incomplete electron transfer within the electron transport chain. This inefficiency leads to the leakage of electrons, which react with oxygen to form superoxide radicals – a primary form of ROS. Under hypoxic conditions, xanthine dehydrogenase is converted to xanthine oxidase, an enzyme that generates superoxide and hydrogen peroxide as byproducts during purine metabolism. These ROS further propagate oxidative damage. Hypoxia stimulates the activity of NADPH oxidase in vascular endothelial cells. This enzyme produces superoxide directly and is a significant contributor to oxidative stress in cerebral vessels. Chronic anemia depletes systemic antioxidant reserves, such as glutathione and superoxide dismutase, which exacerbates the oxidative burden^[5]^.

ROS cause lipid peroxidation, apoptosis, and reduced nitric oxide bioavailability, impairing vasodilation and increasing the risk of ischemic events. ROS disrupt tight junction proteins, increasing vascular permeability and allowing neurotoxic substances to enter the brain. Hypoxia and oxidative stress impair the ability of cerebral blood vessels to adjust blood flow in response to neuronal activity, compounding ischemic damage during strokes. Anemic patients with ischemic strokes tend to present with larger infarct sizes compared to their non-anemic counterparts. This is attributed to the compounded effect of reduced oxygen delivery and impaired vascular regulation. Recovery from stroke is often delayed in anemic individuals. For example, anemic patients in rehabilitation programs frequently show slower progress in regaining motor and cognitive functions, likely due to persistent hypoxia limiting neuronal repair. Population-based studies reveal a 20–30% higher mortality rate among anemic patients with stroke. For instance, a cohort study of stroke patients in North America found that those with moderate to severe anemia (hemoglobin < 10 g/dL) had a significantly higher risk of death within the first 30 days post-stroke. Anemia has been linked to higher rates of post-stroke complications, including infections and cardiac events. This is likely due to systemic hypoxia increasing physiological stress and impairing immune function^[5,6]^.

## Review methodology

### Search strategy

A systematic literature search was conducted using electronic databases, including PubMed, MEDLINE, Embase, and Google Scholar. The search terms used included combinations of keywords related to anemia (e.g., “anemia,” “hemoglobin deficiency”) and cerebrovascular disease (e.g., “stroke,” “cerebral ischemia”). The search was limited to articles published in English and relevant to human studies. Additional articles were identified through manual searches of reference lists and citation tracking.

### Selection criteria

Articles were screened based on predefined inclusion and exclusion criteria. Inclusion criteria encompassed studies examining the relationship between anemia and cerebrovascular disease, including observational studies, clinical trials, systematic reviews, and meta-analyses. Exclusion criteria included studies not directly relevant to the topic, such as animal studies, case reports, and editorials.

### Data extraction

Data extraction was performed independently by two reviewers using a standardized data extraction form. Extracted data included study characteristics (e.g., study design, sample size), participant characteristics (e.g., age, gender), anemia definition and classification, cerebrovascular outcomes (e.g., stroke incidence, cognitive function), and key findings related to the impact of anemia on cerebrovascular health.

### Quality assessment

The quality of included studies was assessed using appropriate tools tailored to the study design. For observational studies, the Newcastle-Ottawa Scale was utilized to assess methodological quality and risk of bias. Randomized controlled trials were evaluated using the Cochrane Collaboration’s tool for assessing risk of bias. Systematic reviews and meta-analyses were assessed using the AMSTAR 2 tool.

## The prevalence of anemia and its impact on cerebrovascular health

Anemia, characterized by a deficiency in red blood cells or hemoglobin, is a global health concern affecting individuals across diverse demographics. While traditionally associated with fatigue and reduced oxygen transport, emerging research suggests a broader impact of anemia on various physiological systems, including its potential influence on cerebrovascular health^[4-8]^. Anemia represents a widespread health issue, affecting people of all ages and socioeconomic backgrounds^[9]^. According to the World Health Organization (WHO), approximately 1.62 billion people globally were affected by anemia in 2016, with the highest prevalence reported in preschool-age children, pregnant women, and individuals over 65 years old. The prevalence varies across regions, with higher rates observed in LMICs^[10]^.

The hallmark consequence of anemia is a reduced capacity to transport oxygen to tissues and organs, including the brain^[11]^. As the primary organ responsible for maintaining vital functions, the brain are particularly sensitive to fluctuations in oxygen levels. In the context of anemia, diminished oxygen-carrying capacity can compromise cerebral oxygenation, potentially leading to adverse cerebrovascular outcomes. Studies have increasingly investigated the correlation between anemia and cerebrovascular complications, with a specific focus on strokes^[12,13]^. Ischemic strokes, resulting from insufficient blood supply to the brain, may be exacerbated by the decreased oxygen-carrying capacity associated with anemia. Hemorrhagic strokes, characterized by bleeding in the brain, may also be influenced by the altered blood viscosity seen in certain types of anemia.

Certain populations are particularly vulnerable to the intersection of anemia and cerebrovascular health^[14]^. For instance, in pregnant women, anemia has been linked to an increased risk of preterm birth and low birth weight, factors that may contribute to long-term cerebrovascular implications for both the mother and the child. Additionally, in older adults, anemia may contribute to cognitive decline, further emphasizing the need to understand the specific implications for this demographic. Chronic conditions, such as chronic kidney disease and inflammatory disorders, often coexist with anemia^[15]^. These conditions not only contribute to the development of anemia but also independently increase the risk of cerebrovascular complications. Recognizing and managing anemia in the context of these comorbidities is crucial for a comprehensive approach to cerebrovascular health. Table 1 shows types of anemia and their impact on cerebrovascular complications.

**Table 1:** Types of anemia and their impact on cerebrovascular complications

Type of Anemia	Mechanism	Association with stroke
Iron deficiency	Reduced oxygen delivery	Increased risk of ischemic stroke
Hemolytic anemia	Endothelial dysfunction	Higher incidence of thrombotic events
Vitamin deficiency	Altered hemodynamics	Linked to hemorrhagic strokes

Anemia remains a global health concern, with its prevalence exhibiting distinct trends and demographic shifts over the past few decades. Despite advances in healthcare and nutrition, anemia affects approximately 1.6 billion people worldwide, representing nearly 24.8% of the global population. The burden is disproportionately high in LMICs, where nutritional deficiencies, infectious diseases, and limited healthcare access exacerbate its prevalence. While efforts to address anemia through fortification programs and maternal-child health interventions have shown some success, certain populations remain at heightened risk, leading to evolving patterns in anemia’s demographics and its impact on cerebrovascular health^[10]^. Between 1990 and 2020, global anemia prevalence declined modestly, particularly among women and children, due to improved nutrition and public health interventions. However, progress has stagnated in recent years, with the WHO reporting that anemia prevalence in children under five remains alarmingly high at 40% in some regions of Africa and South Asia. Anemia remains most prevalent in pregnant and lactating women due to increased iron demands. However, there is growing recognition of its impact on non-pregnant women, particularly those with heavy menstrual bleeding or underlying health conditions. Aging populations in high-income countries have seen a rise in anemia prevalence, often associated with chronic diseases, poor nutrition, or gastrointestinal bleeding. This demographic shift highlights the increasing contribution of anemia to age-related cerebrovascular conditions, including stroke. Anemia in children remains a persistent challenge, particularly in LMICs. Iron deficiency and parasitic infections are significant contributors, with long-term implications for neurodevelopment and cognitive function. Sub-Saharan Africa and South Asia continue to bear the highest anemia burden, with prevalence rates exceeding 50% in some areas. Conversely, high-income countries have lower prevalence rates (around 10–15%) but face unique challenges, such as anemia in aging populations and patients with chronic kidney disease.^[2-11]^

Anemia exerts significant adverse effects on cerebrovascular health, contributing to the pathogenesis and progression of CVD. The interplay of reduced oxygen delivery, hypoxia, and oxidative stress creates a fertile ground for vascular dysfunction, with distinct impacts observed across different populations. Anemia amplifies the severity of ischemic strokes by limiting oxygen delivery to the brain during critical moments. Epidemiological studies have consistently shown that anemic patients experience larger infarct sizes and worse neurological outcomes. For instance, data from the U.S. National Health and Nutrition Examination Survey highlighted a 15–20% higher risk of ischemic stroke in individuals with anemia compared to those without. While less well-studied, anemia has been associated with increased mortality in hemorrhagic stroke, likely due to impaired hemostasis and compromised vascular repair mechanisms. Chronic anemia, even in its milder forms, has been linked to silent brain infarcts and white matter lesions. These subclinical changes, often detected through advanced neuroimaging, are precursors to cognitive decline and dementia, particularly in elderly populations. Anemia during pregnancy increases the risk of pregnancy-related stroke, a leading cause of maternal morbidity and mortality. Age-related anemia worsens cerebral hypoperfusion and accelerates neurodegenerative processes, leading to a higher incidence of vascular cognitive impairment and stroke in this demographic^[13,14]^.

## Mechanisms underlying anemia-related cerebrovascular complications

The relationship between anemia and cerebrovascular complications involves intricate physiological mechanisms that affect oxygen delivery, blood viscosity, and cerebral blood flow regulation^[16]^. Anemia is characterized by a decreased concentration of hemoglobin, the oxygen-carrying component of red blood cells^[17]^. This reduction in hemoglobin levels leads to diminished oxygen-carrying capacity, compromising the ability of the blood to deliver adequate oxygen to the brain. In conditions of anemia, the brain may experience hypoxia, which can trigger a cascade of events, including oxidative stress and inflammation^[18]^. These processes may contribute to the development or exacerbation of cerebrovascular complications.Anemia can influence blood viscosity, a measure of the thickness and stickiness of blood^[19]^. Changes in viscosity can impact blood flow dynamics, potentially affecting the risk of both ischemic and hemorrhagic strokes. Certain types of anemia, such as sickle cell anemia or thalassemia, can lead to abnormal hemoglobin structures and increased blood viscosity. This alteration in viscosity may contribute to the formation of blood clots or impair blood flow through cerebral vessels, predisposing individuals to cerebrovascular events.

Anemia may disrupt the finely tuned regulation of cerebral blood flow, a critical process for maintaining brain function^[20]^. The brain has sophisticated mechanisms to auto-regulate blood flow to meet its metabolic demands. In the context of anemia, impaired oxygen delivery may trigger compensatory mechanisms, such as vasodilation, to enhance blood flow^[21]^. However, chronic anemia can strain these regulatory mechanisms, potentially leading to inadequate perfusion and increasing the vulnerability to ischemic events. Endothelial cells lining blood vessels play a pivotal role in regulating vascular tone and maintaining vascular integrity. Anemia-related hypoxia and inflammation can contribute to endothelial dysfunction, compromising the ability of blood vessels to dilate and constrict appropriately^[22]^. Endothelial dysfunction is associated with atherosclerosis, a major contributor to ischemic strokes. Anemia-induced endothelial dysfunction may amplify the risk of atherosclerotic events in cerebral vessels^[22]^. Anemia-related changes in blood composition and flow dynamics may enhance the propensity for thrombotic events. Platelet aggregation and clot formation may be influenced by altered shear forces and blood viscosity in the context of anemia^[23]^. Thrombotic events can occlude cerebral blood vessels, leading to ischemic strokes. Individuals with anemia, particularly those with additional pro-thrombotic risk factors, may be more susceptible to thromboembolic events. Table 2 shows pathophysiological mechanisms linking anemia to cerebrovascular complications.

**Table 2:** Pathophysiological mechanisms linking anemia to cerebrovascular complications

Mechanism	Description
Hypoxia	Anemia-induced low oxygen levels may lead to cerebral hypoxia, contributing to stroke risk
Inflammation	Chronic inflammation associated with anemia may promote atherosclerosis and thrombosis
Hemodynamic changes	Altered blood flow dynamics in anemic conditions impacting vascular health

## Hypoxia and oxidative stress in the brain

Hypoxia caused by anemia activates hypoxia-inducible factor (HIF)-1α, a transcription factor that upregulates pro-angiogenic and pro-inflammatory genes. While HIF-1α initially aids tissue adaptation by promoting angiogenesis and erythropoiesis, chronic activation in anemia can lead to pathological vascular remodeling and instability. Hypoxia increases the production of ROS from mitochondrial dysfunction and enzymatic pathways like NADPH oxidase and xanthine oxidase. ROS damage endothelial cells, disrupt the blood-brain barrier (BBB), and reduce nitric oxide (NO) bioavailability, impairing vasodilation and exacerbating ischemic injury. Oxidative stress alters endothelial cell function, leading to reduced NO production, increased endothelin-1 (a vasoconstrictor), and enhanced platelet aggregation. These changes heighten the risk of ischemic events by promoting vascular occlusion and inflammation. Anemia-induced hypoxia disrupts the coordination between neuronal activity and blood flow, exacerbating ischemic damage during cerebrovascular events.^[16-18]^

## Anemia types and their impacts on cerebrovascular health

Iron-deficiency anemia (IDA) reduces hemoglobin levels, limiting oxygen transport and causing chronic hypoxia. Iron is also a cofactor for enzymes in mitochondrial respiration; its deficiency exacerbates oxidative stress by impairing cellular metabolism. IDA is associated with increased risks of ischemic stroke, cognitive decline, and silent brain infarcts. Chronic iron deficiency also heightens vulnerability to subclinical cerebrovascular changes, particularly in children and elderly populations. Deficiencies in vitamin B12 or folate impair DNA synthesis, leading to ineffective erythropoiesis and large, dysfunctional red blood cells. The resultant anemia induces hypoxia, and vitamin B12 deficiency also contributes to neurotoxicity through elevated homocysteine levels, which damage endothelial cells and promote atherogenesis. Patients with megaloblastic anemia have an increased risk of large-vessel stroke and small-vessel disease due to endothelial dysfunction and thrombosis. In conditions like autoimmune hemolytic anemia or hereditary spherocytosis, excessive red blood cell breakdown leads to anemia and systemic inflammation. Free hemoglobin released during hemolysis scavenges NO, reducing vasodilation and worsening vascular stress. Hemolytic anemia contributes to microvascular occlusions and ischemia, particularly in the brain, leading to stroke-like symptoms and cognitive impairment.^[19-21]^

## Genetic factors and their distinct mechanisms

Sickle cell anemia (SCA) is caused by a mutation in the β-globin gene, resulting in hemoglobin S, which polymerizes under hypoxic conditions. This leads to red blood cell sickling, vascular occlusion, and hemolysis. Chronic hemolysis further reduces NO bioavailability, exacerbating vasculopathy. Additionally, ischemia-reperfusion injury from transient vascular occlusions amplifies oxidative stress and inflammation. SCA is strongly associated with ischemic and hemorrhagic strokes, silent cerebral infarcts, and moya-moya disease-like vascular abnormalities. Pediatric patients with SCA frequently exhibit neurodevelopmental delays due to recurrent ischemic insults. Thalassemias are caused by mutations in α- or β-globin genes, resulting in ineffective erythropoiesis and chronic anemia. Iron overload from repeated transfusions or increased intestinal absorption generates ROS, leading to oxidative stress and endothelial dysfunction. Patients with thalassemia are at heightened risk of thromboembolic events, cerebrovascular infarcts, and white matter changes, particularly if iron chelation therapy is suboptimal.^[21-23]^

## Pathophysiological insights

### Hemodynamic changes

Anemia significantly affects hemodynamics, altering the way blood circulates and delivers oxygen throughout the body, including the brain. This section explores the compensatory mechanisms and their limits, the impact on cerebral blood flow, and the resultant hypoxic conditions. The primary consequence of anemia is a reduced oxygen-carrying capacity due to a decreased number of red blood cells or low hemoglobin levels. The heart pumps more frequently and with greater force to increase the volume of blood circulating through the body. This elevated cardiac output helps to deliver more oxygen to tissues despite the lower oxygen content per unit of blood. Tissues, including the brain, extract a higher proportion of oxygen from the blood passing through them. Under normal conditions, only about 25-30% of the oxygen in arterial blood is extracted by tissues; this rate increases in anemia. Blood flow is redirected from less vital organs to essential organs like the brain and heart. This prioritization helps maintain oxygen supply to critical areas but can compromise the function of other organs^[16]^.

In response to anemia, the brain increases its blood flow to compensate for reduced oxygen availability. This process, known as cerebral autoregulation, aims to maintain constant cerebral perfusion despite changes in systemic blood pressure and oxygen levels. Initially, the brain increases cerebral blood flow (CBF) to counteract the reduced oxygen-carrying capacity of anemic blood. This hyperemia is a protective response to maintain oxygen delivery. Prolonged anemia can exhaust the body’s compensatory capacity. When the demand for oxygen exceeds the ability of increased blood flow to supply it, cerebral hypoxia ensues. This chronic hypoxia can impair neuronal function and viability. In severe anemia, the combination of insufficient oxygen supply and the inability to maintain adequate blood flow can lead to ischemia. Ischemic conditions exacerbate neuronal injury and increase the risk of cerebrovascular events, such as strokes. Chronic anemia can alter the responsiveness of cerebral blood vessels, impairing their ability to dilate or constrict appropriately. This dysfunction can further compromise cerebral perfusion, particularly during episodes of acute anemia or additional stress. The combination of impaired oxygen delivery, altered blood flow, and vascular dysfunction increases the risk of both ischemic and hemorrhagic strokes. Anemic patients, particularly those with severe or chronic anemia, are at a higher risk for stroke and related complications. Chronic cerebral hypoxia and ischemia can lead to cognitive deficits, ranging from mild cognitive impairment to more severe forms of dementia. The impact of anemia on cognitive function underscores the importance of addressing and managing anemia in at-risk populations. Chronic hypoxia and altered hemodynamics can lead to structural changes in cerebral blood vessels, such as increased vessel wall thickness and reduced elasticity. These changes can further impair blood flow and exacerbate cerebrovascular disease^[17]^.

### Hypoxia and ischemia

Hypoxia and ischemia are central to understanding the impact of anemia on cerebrovascular health. This section delves into the mechanisms by which anemia-induced hypoxia leads to cerebral ischemia and the subsequent effects on neuronal function and survival. Hypoxia occurs when tissues, including the brain, receive inadequate oxygen due to anemia. Hypoxia-inducible factors (HIFs) are transcription factors that regulate the expression of genes involved in the cellular response to low oxygen levels. Under hypoxic conditions, HIFs become stabilized and activate the transcription of genes that promote angiogenesis (formation of new blood vessels), erythropoiesis (production of red blood cells), and metabolic adaptation to hypoxia. To improve oxygen delivery, hypoxia stimulates the growth of new blood vessels. This process is mediated by vascular endothelial growth factor (VEGF), which is upregulated by HIFs. While angiogenesis can enhance oxygen supply, it may not be sufficient to fully compensate for the oxygen deficit in chronic anemia. Hypoxic conditions shift cellular metabolism from aerobic to anaerobic pathways, increasing the production of lactic acid. This metabolic shift is aimed at maintaining ATP production despite low oxygen levels, but it can lead to acidosis and further cellular stress^[18]^.

When the compensatory mechanisms fail to meet the oxygen demands of the brain, ischemia occurs. Ischemia refers to insufficient blood flow to the brain, leading to severe oxygen and nutrient deprivation. Neurons rely heavily on aerobic metabolism for ATP production. Ischemia causes a rapid depletion of ATP, leading to failure of energy-dependent processes such as ion homeostasis. This energy failure results in neuronal depolarization, increased intracellular calcium, and activation of harmful enzymatic pathways. Ischemia-induced energy failure causes the release of excitatory neurotransmitters, particularly glutamate, leading to excitotoxicity. Excessive glutamate activates NMDA receptors, resulting in increased calcium influx and activation of destructive enzymes like proteases, lipases, and endonucleases. This process contributes to neuronal injury and death. Reperfusion, the restoration of blood flow after an ischemic event, can exacerbate neuronal injury through oxidative stress. The sudden influx of oxygen leads to the generation of ROS, which damage cellular components such as lipids, proteins, and DNA. ROS also trigger inflammatory responses, further aggravating tissue damage. Ischemia and subsequent reperfusion activate microglia and astrocytes, the brain’s resident immune cells. These cells release pro-inflammatory cytokines and chemokines, which contribute to secondary injury by promoting further neuronal death and disrupting the BBB. Ischemia compromises the integrity of the BBB, allowing neurotoxic substances and inflammatory cells to penetrate the brain parenchyma. This disruption exacerbates neuronal injury and contributes to the pathogenesis of various cerebrovascular diseases, including stroke and vascular dementia. Anemic patients, especially those with chronic or severe anemia, have a heightened risk of ischemic stroke. The combined effects of hypoxia, oxidative stress, and inflammation significantly increase the likelihood of cerebrovascular events. Chronic hypoxia and ischemia can lead to progressive cognitive decline. Anemic individuals are at higher risk for developing mild cognitive impairment and dementia due to ongoing neuronal injury and dysfunction. Treatments aimed at reducing oxidative stress, controlling inflammation, and protecting the BBB can help mitigate the impact of anemia on cerebrovascular health. Additionally, strategies to improve oxygen delivery, such as optimized management of anemia and promotion of angiogenesis, are critical^[19]^.

### Blood-brain barrier disruption

The blood-brain barrier (BBB) is a critical structure that maintains the brain’s microenvironment by regulating the passage of substances from the bloodstream into the brain. Anemia-induced hypoxia and ischemia can compromise the integrity of the BBB, leading to increased permeability and a host of downstream effects that exacerbate cerebrovascular disease. The tight junctions between endothelial cells restrict the passage of large and potentially harmful molecules, while transport proteins selectively allow necessary nutrients and ions to cross. The BBB ensures a stable environment for neuronal function by regulating the ionic composition and preventing the entry of neurotoxins and pathogens. By limiting the entry of immune cells and inflammatory mediators, the BBB protects the brain from systemic inflammation and autoimmune attacks. Chronic hypoxia stabilizes hypoxia-inducible factors (HIFs), which increase the expression of VEGF. While VEGF promotes angiogenesis, it can also increase BBB permeability by loosening tight junctions between endothelial cells. Ischemia and subsequent reperfusion generate ROS, which damage cellular components including those of the BBB. Oxidative stress disrupts tight junction proteins such as occludin and claudin, leading to increased BBB permeability.Anemia and ischemia activate microglia and astrocytes, releasing pro-inflammatory cytokines (e.g., TNF-α, IL-1β) and chemokines. These inflammatory mediators can further degrade tight junctions and increase BBB permeability. Additionally, they attract leukocytes to the brain, contributing to neuroinflammation. Matrix metalloproteinases (MMPs), which are upregulated in response to hypoxia and inflammation, degrade extracellular matrix components of the BBB. This enzymatic activity weakens the BBB structure, facilitating the entry of potentially harmful substances into the brain^[19]^.

Increased BBB permeability allows neurotoxic substances, such as circulating toxins and excess glutamate, to enter the brain. These substances can cause direct neuronal damage and exacerbate excitotoxicity. The entry of peripheral immune cells and inflammatory mediators into the brain parenchyma amplifies neuroinflammation. Chronic inflammation can lead to progressive neuronal damage and is a key factor in the development of cerebrovascular diseases. BBB disruption can result in vasogenic edema, where fluid leaks into the brain tissue, increasing intracranial pressure and further impairing neuronal function. The compromised BBB exacerbates the damage caused by ischemic strokes. Increased permeability facilitates the entry of blood-borne elements that contribute to secondary brain injury, leading to larger infarct sizes and worse clinical outcomes. Patients with anemia, particularly those with chronic or severe forms, should be monitored for signs of cerebrovascular complications. Early detection and treatment of BBB disruption can help prevent or mitigate the severity of neurological damage. Developing therapies that stabilize the BBB can reduce the impact of anemia-induced cerebrovascular complications. Agents that reduce oxidative stress can protect the BBB from ROS-induced damage. Medications that modulate the inflammatory response can help maintain BBB integrity. Inhibiting matrix metalloproteinases can prevent the degradation of the BBB’s extracellular matrix.Effective management of anemia itself, through iron supplementation, erythropoiesis-stimulating agents, or other treatments, can reduce hypoxia and its downstream effects on the BBB^[20]^.

### Anemia-induced changes and their impact on BBB function

Anemia, particularly when severe or chronic, induces a state of hypoxia due to reduced oxygen-carrying capacity in the blood. In anemia, the lack of adequate oxygen supply to brain tissue triggers a cascade of compensatory mechanisms, such as the activation of HIF-1α. While HIF-1α initially stimulates angiogenesis and erythropoiesis, prolonged activation due to chronic anemia can cause pathological vascular remodeling. This remodeling leads to an increased permeability of the blood-brain barrier. Hypoxia increases the secretion of VEGF, a potent promoter of angiogenesis and vascular permeability. In the context of anemia, elevated VEGF levels can lead to endothelial cell dysfunction, compromising the BBB’s structural integrity. This allows for the leakage of harmful molecules and immune cells into the brain, further exacerbating damage and promoting neuroinflammation. Anemia-induced hypoxia and increased ROS production further damage the endothelial cells that line the blood-brain barrier. ROS can degrade tight junction proteins such as occludins and claudins, which are responsible for maintaining BBB integrity. This degradation results in the loss of selective permeability, allowing potentially harmful substances to enter the brain parenchyma. As the BBB becomes more permeable, inflammatory mediators like cytokines (e.g., TNF-α, IL-6) and chemokines are able to pass through, initiating a cascade of neuroinflammatory responses. These inflammatory signals recruit immune cells such as microglia and leukocytes, which can further damage the BBB and surrounding neural tissue. Chronic neuroinflammation can impair neuronal function and exacerbate cognitive decline, especially in populations with pre-existing vascular risk factors or genetic predispositions^[19,20]^.

## Reperfusion injury and its exacerbation of BBB disruption

Following an ischemic event, the restoration of blood flow (reperfusion) is intended to salvage tissue and restore oxygen supply to ischemic regions of the brain. However, reperfusion can paradoxically lead to further damage, particularly through the exacerbation of BBB disruption. This phenomenon, known as reperfusion injury, is particularly relevant in patients with anemia who already have compromised cerebral perfusion prior to ischemia. When blood flow is restored to ischemic brain tissue, the sudden influx of oxygen leads to a burst of ROS production, overwhelming the antioxidant defenses in the brain. These ROS further damage endothelial cells of the BBB, disrupting tight junctions and promoting endothelial cell apoptosis. The loss of tight junction integrity increases the permeability of the BBB, allowing plasma proteins, leukocytes, and toxic metabolites to infiltrate the brain tissue, exacerbating ischemic injury. Reperfusion injury also triggers a systemic inflammatory response that further amplifies neuroinflammation. The reperfusion phase mobilizes inflammatory cells such as neutrophils and macrophages, which, upon entering the brain, release cytokines and proteases that degrade the extracellular matrix, further compromising the BBB. Inflammatory mediators such as MMPs are activated during reperfusion, leading to the breakdown of the blood-brain barrier and exacerbating neuronal injury. Reperfusion injury is closely associated with the onset of neuroinflammation, which plays a key role in the progression of secondary brain injury following ischemia. As the BBB becomes more permeable, inflammatory cells (including neutrophils and monocytes) infiltrate the brain parenchyma. The presence of these cells, along with pro-inflammatory cytokines, further disrupts the BBB, creating a vicious cycle of BBB breakdown and neuronal damage. Reperfusion also activates microglia, the resident immune cells of the central nervous system. These cells release inflammatory cytokines, which exacerbate neuronal injury, promote oxidative stress, and continue to damage the already compromised BBB. Chronic activation of microglia leads to prolonged neuroinflammation, which can impair neurovascular coupling and increase the risk of chronic cerebrovascular diseases, including stroke and vascular dementia.^[18-20]^

### Consequences of BBB disruption in anemic patients post-ischemia

In patients with anemia, the pre-existing hypoxic and oxidative stress conditions further compound the deleterious effects of ischemia and reperfusion injury. This dual insult significantly impacts the integrity of the BBB and accelerates brain damage. The increased permeability of the BBB allows for the extravasation of plasma proteins, water, and electrolytes into the brain parenchyma, contributing to cerebral edema. In the context of anemia, this can worsen brain swelling and lead to increased intracranial pressure, which further reduces cerebral perfusion and exacerbates ischemic injury. Chronic BBB disruption and neuroinflammation in anemic patients can result in long-term cognitive impairment and neurodegeneration. The leakage of inflammatory cells and toxic molecules into the brain accelerates neuronal loss, particularly in regions like the hippocampus and cortex, which are critical for memory and cognitive function. This is particularly concerning in populations such as the elderly, who are already at risk for age-related neurodegenerative diseases. Anemia-induced BBB disruption, in combination with ischemic and reperfusion injury, significantly increases the risk of stroke. Even subclinical ischemic events (silent infarcts) can be exacerbated by a compromised BBB, leading to a cascade of microvascular damage and further ischemic episodes. Over time, this contributes to vascular cognitive impairment, particularly in patients with chronic anemia or recurrent strokes^[19,20]^.

### Inflammatory responses

Anemia is often associated with a pro-inflammatory state, which plays a significant role in the pathophysiology of cerebrovascular diseases. This section explores how anemia-induced inflammation contributes to cerebrovascular complications, focusing on cellular and molecular mechanisms and their clinical implications. Anemia can stimulate the production of pro-inflammatory cytokines such as tumor necrosis factor-alpha (TNF-α), interleukin-1 beta (IL-1β), and interleukin-6 (IL-6). These cytokines are released by activated immune cells, including macrophages and microglia, in response to hypoxic and ischemic conditions. Hypoxia and ischemia resulting from anemia activate resident immune cells in the brain, such as microglia and astrocytes. These cells become reactive, releasing additional pro-inflammatory mediators that amplify the inflammatory response. Anemia-induced hypoxia increases the production of ROS, which not only cause direct cellular damage but also act as signaling molecules to further stimulate inflammatory pathways. ROS activate transcription factors like nuclear factor-kappa B (NF-κB), which upregulate the expression of inflammatory genes. Hypoxia and oxidative stress impair endothelial cell function, promoting the expression of adhesion molecules like vascular cell adhesion molecule-1 (VCAM-1) and intercellular adhesion molecule-1 (ICAM-1). These molecules facilitate the adhesion and transmigration of leukocytes across the BBB, enhancing neuroinflammation^[21]^.

Chronic inflammation damages the endothelial lining of blood vessels, promoting the development of atherosclerosis. Inflammatory cytokines stimulate the proliferation of smooth muscle cells and the deposition of extracellular matrix, leading to plaque formation. These atherosclerotic plaques can rupture, causing thromboembolic events that increase the risk of ischemic stroke. Inflammatory mediators such as cytokines and ROS disrupt the integrity of the BBB. Increased permeability allows the infiltration of immune cells and neurotoxic substances into the brain parenchyma, exacerbating neuronal injury and contributing to the pathogenesis of stroke and other cerebrovascular diseases. Activated microglia and astrocytes release pro-inflammatory cytokines, chemokines, and ROS, creating a toxic environment that promotes neuronal death. Chronic neuroinflammation can lead to neurodegenerative changes and cognitive impairment. Inflammation promotes a hypercoagulable state by increasing the expression of tissue factor and reducing the levels of anticoagulant proteins. This pro-thrombotic environment heightens the risk of thromboembolic events, which are common precursors to ischemic strokes. Recognizing the inflammatory component of anemia can improve risk stratification for cerebrovascular diseases. Patients with chronic anemia should be monitored for inflammatory markers, as elevated levels may indicate an increased risk of cerebrovascular complications. Targeting inflammation offers a potential therapeutic strategy to mitigate the impact of anemia on cerebrovascular health. Anti-inflammatory drugs such as corticosteroids, non-steroidal anti-inflammatory drugs (NSAIDs), and specific cytokine inhibitors (e.g., anti-TNF agents) can reduce inflammation and protect the cerebrovascular system. Antioxidants that reduce oxidative stress can help manage the inflammatory response associated with anemia. Agents such as N-acetylcysteine (NAC), vitamin E, and other ROS scavengers can protect endothelial cells and neurons from oxidative damage. Effective treatment of anemia, including iron supplementation and the use of erythropoiesis-stimulating agents, can reduce hypoxia-induced inflammation. Optimizing hemoglobin levels decreases the production of pro-inflammatory mediators and improves overall cerebrovascular health^[22]^.

### Oxidative stress

Oxidative stress plays a significant role in the pathophysiology of anemia-related cerebrovascular complications. In anemia, hypoxia leads to increased production of ROS such as superoxide anions, hydrogen peroxide, and hydroxyl radicals. These ROS are generated primarily in the mitochondria during cellular respiration when oxygen levels are insufficient. Under normal conditions, the body’s antioxidant defenses, including enzymes like superoxide dismutase (SOD), catalase, and glutathione peroxidase, neutralizes ROS. Anemia-induced hypoxia overwhelms these antioxidant systems, leading to an accumulation of ROS. As discussed previously, anemia-induced inflammation further amplifies oxidative stress. Activated immune cells, such as microglia and macrophages, produce additional ROS and pro-inflammatory cytokines, creating a vicious cycle of oxidative damage and inflammation. Iron, a key component of hemoglobin, plays a central role in oxidative stress. In conditions of iron deficiency or overload, free iron catalyzes the Fenton reaction, producing highly reactive hydroxyl radicals. These radicals cause significant cellular damage and contribute to oxidative stress. ROS directly damage endothelial cells lining the cerebral blood vessels. This damage disrupts the integrity of the endothelial barrier, leading to increased vascular permeability and promoting atherosclerosis. Endothelial dysfunction impairs vasodilation, exacerbating ischemic conditions in the brain. Oxidative stress compromises the BBB by degrading tight junction proteins such as occludin and claudin. The increased permeability of the BBB allows harmful substances, including inflammatory cells and neurotoxins, to infiltrate the brain, exacerbating neuronal injury. Neurons are particularly vulnerable to oxidative stress due to their high metabolic rate and limited antioxidant defenses. ROS damage neuronal membranes, proteins, and DNA, leading to cell death. Oxidative stress also impairs mitochondrial function, further depleting cellular energy stores and exacerbating neuronal injury. Oxidative stress contributes to excitotoxicity, a process where excessive glutamate release and receptor activation lead to neuronal death. ROS enhance glutamate release and impair the reuptake of glutamate by astrocytes, prolonging excitotoxic signaling and increasing neuronal damage. ROS act as signaling molecules that activate transcription factors such as nuclear factor-kappa B (NF-κB), which upregulate the expression of pro-inflammatory genes. This oxidative-inflammatory feedback loop perpetuates chronic neuroinflammation, contributing to the progression of cerebrovascular diseases and cognitive decline^[23]^.

Recognizing oxidative stress as a key factor in anemia-related cerebrovascular complications can improve risk stratification. Measuring biomarkers of oxidative stress, such as malondialdehyde (MDA) and 8-hydroxy-2ʹ-deoxyguanosine (8-OHdG), can help identify patients at higher risk for stroke and other cerebrovascular events. Targeting oxidative stress with antioxidant therapies offers a potential approach to mitigate cerebrovascular complications in anemic patients. Antioxidants such as N-acetylcysteine (NAC), vitamin C, vitamin E, and coenzyme Q10 can scavenge ROS and enhance endogenous antioxidant defenses. Since inflammation and oxidative stress are closely linked, anti-inflammatory treatments can also help reduce oxidative damage. Drugs such as corticosteroids, non-steroidal anti-inflammatory drugs (NSAIDs), and specific cytokine inhibitors can attenuate the oxidative-inflammatory cascade. Proper management of iron levels are crucial in anemic patients. Iron supplementation should be carefully monitored to avoid iron overload, which can exacerbate oxidative stress. In cases of iron deficiency anemia, appropriate iron supplementation can help restore normal hemoglobin levels and reduce hypoxia-induced oxidative stress. Addressing the underlying causes of anemia through treatments like erythropoiesis-stimulating agents, iron supplementation, and nutritional support can reduce hypoxia and its associated oxidative stress. Improving oxygen delivery to tissues can mitigate the cascade of oxidative damage and improve cerebrovascular outcomes^[15]^.

### Sources and mechanisms of ROS production beyond mitochondria

The endoplasmic reticulum is a key organelle responsible for protein folding and maintaining cellular homeostasis. Under stress conditions, such as those seen in anemia, the ER can become overwhelmed with misfolded proteins, triggering a response known as the unfolded protein response (UPR). When the UPR is prolonged or dysregulated, it can lead to the excessive production of ROS. The ER is rich in proteins that contain disulfide bonds, which are essential for protein structure. When protein folding is impaired, misfolded proteins accumulate, leading to ER stress. This stress activates the NADPH oxidase (NOX) enzymes in the ER membrane, which are potent sources of ROS. Additionally, the accumulation of calcium within the ER can activate redox-sensitive enzymes, which further promote the generation of ROS within the organelle. In anemic states, particularly when hemoglobin synthesis is impaired (e.g., in iron-deficiency anemia), the ER is under increased stress due to the accumulation of misfolded hemoglobin chains. This results in the activation of ROS-producing pathways in the ER, contributing to cellular damage and inflammation. NADPH oxidases are enzymes found in various cellular compartments, including the plasma membrane, ER, and endosomes. These enzymes are significant contributors to ROS production, particularly in the presence of inflammation or cellular stress. In cells such as endothelial cells, NOX enzymes produce ROS by transferring electrons from NADPH to oxygen. During inflammatory conditions, such as anemia, the activation of NOX enzymes in the plasma membrane is heightened, resulting in the generation of superoxide anions, which can further damage cellular structures. The NOX enzyme complex is also present in endosomes, where it produces ROS during phagocytosis. In the context of anemia, particularly hemolytic anemia, the phagocytic activity of macrophages is heightened, leading to increased ROS production through NOX enzymes. Peroxisomes are involved in various metabolic processes, including lipid metabolism and the detoxification of reactive molecules. These organelles also play a significant role in ROS production. Peroxisomes contain enzymes such as fatty acid oxidase and catalase that, during metabolic stress, can generate ROS, particularly hydrogen peroxide (H₂O₂). In anemia, where oxidative stress is already elevated due to insufficient oxygen delivery, the activity of peroxisomal enzymes can exacerbate ROS accumulation, further contributing to cellular damage. Lysosomes, the cellular organelles responsible for degrading waste materials, can also be a source of ROS. Under certain stress conditions, such as those seen in anemia, lysosomal degradation of cellular components can lead to the release of ROS. When cellular components are degraded or when lysosomal membrane stability is compromised, the lysosome releases iron from heme degradation. This free iron can catalyze ROS production via the Fenton reaction, where iron reacts with hydrogen peroxide to produce hydroxyl radicals, potent oxidants that can damage cellular components.^[19-22]^

### Regulation of iron homeostasis and disruptions in anemia

Iron plays a central role in ROS production due to its involvement in the Fenton reaction. In this reaction, iron (Fe^2^⁺) reacts with hydrogen peroxide (H₂O₂) to generate highly reactive hydroxyl radicals (OH•). Under normal conditions, iron homeostasis is tightly regulated to prevent excessive iron buildup, which can lead to toxic ROS generation. However, in anemia, disturbances in iron metabolism contribute significantly to the oxidative stress that exacerbates tissue injury. In normal physiology, iron is stored in ferritin and transported in the blood by transferrin. The body regulates iron levels by controlling absorption in the intestines and ensuring proper recycling by macrophages. In iron-deficiency anemia, the body has reduced iron stores, but the iron that is present may be inadequately incorporated into hemoglobin or other heme-containing proteins. This leads to inefficient oxygen delivery to tissues and compensatory mechanisms such as increased erythropoiesis. However, iron accumulation in the tissues, due to the limited ability to properly incorporate iron into hemoglobin, can lead to free iron being released into the bloodstream. Free iron in excess can catalyze the production of ROS, particularly in tissues such as the brain and vasculature, where iron overload is particularly damaging. In contrast, anemia of chronic disease is characterized by impaired iron release from macrophages and decreased iron availability for erythropoiesis. The resulting iron sequestration in macrophages, while limiting the availability of free iron, can still contribute to oxidative stress. This occurs as iron remains in storage pools (such as ferritin), and the release of ROS from macrophages is still upregulated, albeit in different patterns compared to iron overload situations. In hemolytic anemia, the premature destruction of red blood cells releases large amounts of hemoglobin, which contains iron. The free heme from the lysed red blood cells can also contribute to oxidative stress by promoting ROS production in the bloodstream and tissues. This unregulated iron release significantly increases the chances of ROS formation, which can lead to endothelial damage and exacerbate conditions such as cardiovascular disease and stroke. Iron that is not bound to hemoglobin or ferritin is highly reactive and can catalyze the generation of ROS via the Fenton reaction. This free iron is more likely to accumulate in tissues, where it can cause direct cellular damage, particularly in organs like the liver, kidneys, and brain, increasing vulnerability to neurovascular complications. As red blood cells are destroyed, hemoglobin is broken down into heme, which contains iron. This heme is then further metabolized in macrophages, and its degradation can release iron into the circulation. This free iron can contribute to oxidative stress and inflammation, particularly in the brain, where it may impair the function of the blood-brain barrier and exacerbate neurological damage.^[21-23]^

### Endothelial dysfunction

Endothelial dysfunction is a pivotal factor linking anemia to cerebrovascular disease. The endothelium, the inner lining of blood vessels, plays a crucial role in vascular homeostasis, regulating blood flow, coagulation, and immune response. Anemia-induced hypoxia and oxidative stress significantly impair endothelial function, leading to a cascade of pathological events. Nitric oxide, produced by endothelial cells, is essential for vasodilation and maintaining vascular tone. Hypoxia and oxidative stress in anemia reduce NO bioavailability by increasing the production of ROS, which react with NO to form peroxynitrite, a potent oxidant that further depletes NO levels. Anemia-induced hypoxia enhances mitochondrial ROS production. Excessive ROS damage endothelial cells directly, disrupt cell membranes, and degrade essential proteins, including those involved in maintaining tight junctions and vascular integrity. Hypoxia and oxidative stress activate transcription factors like nuclear factor-kappa B (NF-κB), which upregulate the expression of pro-inflammatory cytokines (e.g., TNF-α, IL-1β) and adhesion molecules (e.g., VCAM-1, ICAM-1). These molecules facilitate leukocyte adhesion and migration into the vessel wall, promoting inflammation and endothelial dysfunction. The structural integrity of the endothelium is maintained by tight junction proteins such as occludin, claudin, and zonula occludens-1 (ZO-1). Oxidative stress and inflammation lead to the degradation of these proteins, increasing endothelial permeability and compromising the BBB. Hypoxia-induced factors like VEGF promote angiogenesis to improve oxygen delivery. However, chronic overexpression of VEGF can disrupt the endothelial barrier, increasing permeability and contributing to vascular leakage and edema^[18]^.

Reduced NO availability and endothelial cell damage impair vasodilation, leading to increased vascular resistance and reduced cerebral blood flow. This contributes to ischemic conditions and exacerbates hypoxia in the brain. Endothelial dysfunction promotes the development of atherosclerotic plaques by increasing the adhesion of monocytes and their transformation into foam cells. Chronic inflammation and oxidative stress contribute to plaque formation, progression, and instability, increasing the risk of thromboembolic events and ischemic stroke. The increased permeability of the endothelial barrier allows neurotoxic substances and inflammatory cells to enter the brain parenchyma. This contributes to neuroinflammation, neuronal damage, and the progression of cerebrovascular diseases such as stroke and vascular dementia. Endothelial dysfunction disrupts the balance between pro-coagulant and anti-coagulant factors. Anemia-induced hypoxia increases the expression of tissue factor and reduces the production of anticoagulant molecules like thrombomodulin, promoting a pro-thrombotic state and increasing the risk of cerebrovascular events. Assessing markers of endothelial dysfunction, such as flow-mediated dilation (FMD), levels of endothelial microparticles, and biomarkers like VCAM-1, ICAM-1, and E-selectin, can help identify patients at risk for cerebrovascular complications. Antioxidants can mitigate oxidative stress and protect endothelial cells. Agents like N-acetylcysteine (NAC), vitamin C, vitamin E, and polyphenols can scavenge ROS and support endothelial function. Reducing inflammation with medications such as corticosteroids, non-steroidal anti-inflammatory drugs (NSAIDs), and specific cytokine inhibitors (e.g., anti-TNF agents) can help preserve endothelial integrity and function. Therapies that increase NO availability, such as NO donors (e.g., nitroglycerin) or phosphodiesterase inhibitors (e.g., sildenafil), can improve endothelial function and vasodilation. Effective treatment of anemia, including iron supplementation, erythropoiesis-stimulating agents, and nutritional support, can alleviate hypoxia and reduce its detrimental effects on the endothelium. Optimizing hemoglobin levels improves oxygen delivery and endothelial health. Encouraging patients to adopt healthy lifestyle practices, such as regular exercise, a balanced diet rich in antioxidants, and smoking cessation, can support endothelial function and reduce the risk of cerebrovascular disease^[19]^.

### Neurovascular coupling

Neurovascular coupling refers to the relationship between neuronal activity and CBF. It is essential for maintaining brain function and health, ensuring that regions with higher activity receive adequate oxygen and nutrients. Anemia, through various mechanisms, can disrupt this critical process, leading to cerebrovascular complications. This section explores how anemia affects neurovascular coupling and its clinical implications. Neuronal activity increases the metabolic demand for oxygen and glucose. Astrocytes and other glial cells detect this increased activity and release vasoactive substances that dilate blood vessels, increasing local blood flow. Endothelial cells play a crucial role in neurovascular coupling by producing NO, a potent vasodilator. NO production is stimulated by increased shear stress and metabolic signals from active neurons.Astrocytes, which bridge neurons and blood vessels, release vasodilatory signals such as potassium ions (K+), adenosine, and arachidonic acid metabolites (e.g., prostaglandins) in response to neuronal activity. These signals help modulate blood flow to match metabolic needs. The neurovascular unit comprises neurons, astrocytes, endothelial cells, pericytes, and smooth muscle cells. Coordination among these cells ensures the precise regulation of CBF in response to neuronal activity. Anemia decreases the oxygen-carrying capacity of blood, leading to chronic cerebral hypoxia. This impairs the brain’s ability to meet the metabolic demands of active neurons, disrupting the balance of oxygen supply and demand. Hypoxia and oxidative stress in anemia reduce NO bioavailability by enhancing the production of ROS, which scavenge NO. This leads to diminished vasodilatory responses and inadequate blood flow to active brain regions. Oxidative stress and inflammation associated with anemia can impair astrocyte function, reducing their ability to release vasodilatory signals. This further compromises the neurovascular coupling process.As previously discussed, anemia-induced endothelial dysfunction leads to impaired vasodilation and increased vascular resistance. This disrupts the dynamic regulation of blood flow necessary for efficient neurovascular coupling. Chronic inflammation in anemia upregulates pro-inflammatory cytokines, which can alter the function of the neurovascular unit. Inflammatory mediators can disrupt signaling pathways between neurons, astrocytes, and endothelial cells, impairing the coordination of CBF^[16]^.

Disrupted neurovascular coupling in anemia can lead to cognitive deficits. Insufficient blood flow to active brain regions impairs neuronal function and connectivity, contributing to cognitive decline and increasing the risk of conditions like vascular dementia. Impaired neurovascular coupling reduces the brain’s ability to regulate blood flow effectively, increasing susceptibility to ischemic events. Anemia-related endothelial dysfunction and pro-thrombotic states further exacerbate this risk.Recognizing the impact of anemia on neurovascular coupling can improve the diagnosis and management of cerebrovascular diseases. Advanced imaging techniques, such as functional MRI (fMRI) and near-infrared spectroscopy (NIRS), can assess neurovascular coupling and identify areas of impaired CBF. Addressing the underlying causes of impaired neurovascular coupling can improve cerebrovascular outcomes in anemic patients. Potential strategies include:Reducing oxidative stress can enhance NO availability and improve endothelial function. Antioxidants such as N-acetylcysteine (NAC), vitamin C, and vitamin E can support neurovascular health. Reducing inflammation with corticosteroids, NSAIDs, or specific cytokine inhibitors can help restore the function of the neurovascular unit. Drugs that protect neuronal and glial cells from hypoxic and oxidative damage can improve neurovascular coupling. Examples include erythropoietin (EPO) and other neurotrophic factors. Effective treatment of anemia through iron supplementation, erythropoiesis-stimulating agents, and nutritional support can improve oxygen delivery and support neurovascular health^[20]^.

### Anemia as a risk factor for stroke

Stroke, a significant cause of morbidity and mortality worldwide, is characterized by the sudden interruption of blood flow to the brain, leading to neurological impairment^[24]^. While traditional risk factors for stroke such as hypertension and diabetes are well-established, emerging evidence suggests that anemia may represent an independent risk factor for the occurrence and severity of strokes. Numerous epidemiological studies and meta-analyses have investigated the association between anemia and the risk of stroke^[25]^. These investigations encompass diverse populations, including different age groups, ethnicities, and geographical regions. Findings consistently suggest that anemic individuals may face a higher risk of experiencing both ischemic and hemorrhagic strokes compared to non-anemic counterparts. The relationship between anemia and stroke risk appears to be influenced by the specific type and severity of anemia^[26]^. For instance, iron-deficiency anemia, the most common form of anemia worldwide, has been particularly implicated in ischemic strokes due to its association with increased blood viscosity and reduced oxygen-carrying capacity. Additionally, severe forms of anemia may amplify the risk, highlighting the importance of considering both the type and severity in assessing stroke susceptibility.

Anemia, characterized by low hemoglobin levels, compromises the blood’s ability to carry oxygen^[27]^. This reduction in oxygen-carrying capacity may contribute to ischemic strokes, where a lack of oxygen leads to damage in the brain tissue. Certain types of anemia, such as iron-deficiency anemia, can increase blood viscosity^[28]^. Elevated viscosity may impede normal blood flow, potentially contributing to the development of thrombi and increasing the risk of ischemic strokes. Anemia may disrupt the delicate regulation of cerebral blood flow, impacting the brain’s ability to maintain optimal perfusion. This dysregulation can influence both ischemic and hemorrhagic stroke risk^[28]^. Recognizing anemia as a potential risk factor for stroke holds significant clinical implications. Healthcare practitioners should consider evaluating anemic patients for their stroke risk, especially in populations with a high prevalence of anemia. Additionally, addressing and managing anemia may become a critical component of stroke prevention strategies, potentially mitigating the impact of this modifiable risk factor.

### Clinical implications and management strategies

Recognizing the relationship between anemia and cerebrovascular complications, particularly strokes, holds significant clinical implications for healthcare practitioners. Healthcare practitioners should incorporate anemia assessment into routine stroke risk evaluations. Anemia, given its association with impaired oxygen delivery and altered blood viscosity, may contribute to the development or exacerbation of cerebrovascular events^[29]^. Certain populations, such as the elderly and individuals with chronic diseases, may be more susceptible to the intersection of anemia and cerebrovascular complications. Targeted screening programs for anemia in these high-risk groups can aid in early detection and intervention. Anemic patients should undergo a thorough evaluation, considering the type and severity of anemia, underlying causes, and associated comorbidities^[20]^. Understanding the specific context of anemia is essential for tailoring management strategies. Table 3 shows clinical manifestations of cerebrovascular complications in anemic patients.

**Table 3:** Clinical manifestations of cerebrovascular complications in anemic patients

Manifestation	Frequency in anemic patients	Characteristics
Ischemic stroke	70%	Often associated with atherosclerosis in anemic individuals
Hemorrhagic stroke	30%	Higher prevalence in certain types of anemia, such as vitamin deficiency

The primary goal in managing anemic patients to prevent cerebrovascular complications is to optimize hemoglobin levels. Iron supplementation, erythropoiesis-stimulating agents, or other relevant interventions should be considered based on the underlying cause and type of anemia. Anemia often results from underlying conditions, such as nutritional deficiencies, chronic diseases, or gastrointestinal bleeding^[30]^. Identifying and treating these underlying causes not only addresses the anemia but also contributes to overall cerebrovascular health. Therapeutic approaches should be tailored based on the type of anemia. For instance, in cases of iron-deficiency anemia, iron supplementation may be the primary intervention, while vitamin supplementation or immunosuppressive therapies may be warranted for other types of anemia. Given the potential impact of anemia on blood viscosity and cerebral blood flow, managing blood pressure and vascular health is crucial. This includes optimizing hypertension control and addressing other vascular risk factors^[28]^. Multidisciplinary collaboration is essential for comprehensive anemia management. In cases where anemia is related to chronic diseases, collaboration with specialists in nephrology, gastroenterology, or rheumatology may be necessary for a holistic approach to patient care. Table 4 shows management strategies for anemic patients at risk of cerebrovascular complications.

**Table 4:** Management strategies for anemic patients at risk of cerebrovascular complications

Strategy	Description
Iron supplementation	Correcting iron deficiency to improve oxygen-carrying capacity
Antiplatelet therapy	Consideration of antiplatelet agents in anemic patients with high thrombotic risk
Comprehensive care	Multidisciplinary approach involving neurologists and hematologists for optimal patient management

### Screening tools and guidelines for assessing anemia in the context of cerebrovascular risk

Given the widespread impact of anemia on cerebrovascular health, early screening and prompt identification are essential in preventing adverse outcomes. Several tools and guidelines exist to assess anemia, with a focus on cerebrovascular risk.

1. Routine hemoglobin measurement and hematocrit levels

The first-line approach to detecting anemia is through routine blood tests, including hemoglobin and hematocrit measurements. Hemoglobin levels below 12 g/dL in women and 13 g/dL in men typically indicate anemia. In patients with cerebrovascular disease, especially those presenting with symptoms of hypoperfusion or cognitive decline, this simple measurement serves as a screening tool for anemia that may exacerbate cerebrovascular dysfunction. However, this initial screening test is not sufficient on its own to assess the severity of the disease or its underlying cause^[27]^.

2. Red cell indices and reticulocyte count

Further laboratory investigations, such as mean corpuscular volume (MCV), mean corpuscular hemoglobin (MCH), and reticulocyte count, provide important insights into the type and cause of anemia. Anemia characterized by microcytic cells (low MCV) suggests iron deficiency or thalassemia, while macrocytic cells (high MCV) could indicate vitamin B12 or folate deficiencies. Reticulocyte counts help assess bone marrow response to anemia, indicating whether the anemia is regenerative (active response) or hypoproliferative (due to inadequate erythropoiesis)^[28]^.

3. Peripheral blood smear

A peripheral blood smear remains an essential diagnostic tool, offering visual insights into the morphology of red blood cells. In anemia patients with cerebrovascular risk, the presence of abnormal cell shapes such as spherocytes (suggestive of hemolytic anemia) or sickle cells (in sickle cell anemia) provides valuable clues for further investigation and management^[29]^.

4. Guidelines for screening in high-risk populations

Current guidelines, such as those provided by the WHO and the National Institutes of Health, recommend routine screening for anemia in high-risk groups, including the elderly, pregnant women, individuals with chronic diseases (such as chronic kidney disease, heart disease, and inflammatory disorders), and those with known cerebrovascular risk factors. In cerebrovascular disease patients, regular screening for anemia is crucial, as anemia can contribute to poor prognosis, cognitive decline, and impaired functional recovery^[20]^.

### Advanced diagnostic techniques for understanding the underlying causes of anemia

For patients with cerebrovascular disease, understanding the underlying causes of anemia is essential to tailoring appropriate interventions. Advanced diagnostic techniques can help identify the root causes of anemia, which may be multifactorial or related to specific pathologies that directly impact cerebrovascular health.

1. Bone marrow biopsy

A bone marrow biopsy is an invasive procedure used to assess bone marrow function and determine whether anemia is caused by inadequate erythropoiesis or a problem with hematopoietic stem cells. In patients with cerebrovascular risk factors and unexplained anemia, a bone marrow biopsy can reveal conditions such as myelodysplastic syndromes, leukemia, or bone marrow infiltration by metastases, which may affect both blood cell production and cerebrovascular health. For example, anemia caused by marrow failure or malignancy can lead to both reduced oxygen-carrying capacity and impaired blood-brain barrier function, which are critical in managing cerebrovascular disease outcomes^[30]^.

2. Genetic testing for hemoglobinopathies and thalassemia

Genetic testing has become a powerful tool for diagnosing inherited forms of anemia, such as sickle cell disease, thalassemia, and other hemoglobinopathies. These conditions are particularly important in cerebrovascular disease, as patients with SCA are at a higher risk of cerebrovascular events, including stroke, due to the occlusion of blood vessels by sickle-shaped red blood cells. Early identification of these genetic disorders allows for tailored management strategies, such as regular blood transfusions for patients with sickle cell disease to reduce stroke risk, or iron chelation therapy for those with thalassemia to manage iron overload, a potential source of oxidative stress and further vascular damage^[20]^.

3. Bone marrow iron studies

In cases of suspected iron deficiency anemia, bone marrow iron studies can provide additional insight into iron stores within the body. These studies involve evaluating bone marrow aspirates for iron staining and assessing the amount of iron available for erythropoiesis. Understanding iron stores is particularly relevant in anemia-related cerebrovascular disease because inadequate iron can impair the oxygen-carrying capacity of red blood cells, worsening hypoxia in brain tissues. Conversely, iron overload can exacerbate oxidative stress, contributing to endothelial dysfunction and compromising the blood-brain barrier, making this information valuable for therapeutic decisions^[31]^.

4. Advanced imaging techniques

Advanced imaging techniques, such as MRI with contrast or magnetic resonance angiography (MRA), can be useful in evaluating cerebrovascular changes in patients with anemia. These techniques can detect subtle ischemic changes in the brain, such as microinfarctions or hypoperfusion, which are particularly important in understanding how anemia-induced hypoxia may contribute to long-term cognitive deficits and stroke risk. In patients with sickle cell anemia or thalassemia, imaging can help identify early vascular changes that predispose individuals to cerebrovascular events^[32]^.

### Management strategies for anemia in cerebrovascular disease

Once anemia is diagnosed and its underlying cause is identified, targeted management strategies are critical to improving outcomes for patients with cerebrovascular risk. The management approach should be individualized, addressing the specific type of anemia and the patient’s cerebrovascular health status.

1. Correction of anemia

The first step in managing anemia in cerebrovascular patients is to correct the underlying anemia. This could involve:

- Iron supplementation: For iron-deficiency anemia, oral or intravenous iron supplementation is commonly used to replenish iron stores and restore normal erythropoiesis. In cases where there is significant blood loss or chronic iron loss, iron infusion therapy may be necessary^[29]^.
- Vitamin and mineral replacement: Megaloblastic anemias due to vitamin B12 or folate deficiencies can be corrected with appropriate supplementation, often through injections or high-dose oral supplements^[20]^.
- Erythropoiesis-stimulating agents (ESAs): In cases of anemia due to chronic kidney disease or other causes of impaired erythropoiesis, ESAs such as erythropoietin can stimulate the production of red blood cells.
- Blood transfusions: In cases of severe anemia or when anemia is linked to hemolytic conditions, blood transfusions may be necessary to stabilize the patient, particularly in individuals with sickle cell disease, thalassemia, or other hemoglobinopathies^[20]^.

2. Management of underlying conditions

Addressing the root cause of anemia, such as chronic disease, nutritional deficiencies, or genetic disorders, is essential for preventing recurrence. In patients with thalassemia or other disorders causing iron overload, chelation therapy is important to prevent iron toxicity, which can damage organs, including the brain, and worsen cerebrovascular outcomes. In sickle cell disease, hydroxyurea therapy can help reduce the frequency of vaso-occlusive events, thus minimizing the risk of strokes and other cerebrovascular complications^[20,30]^.

3. Cerebrovascular disease management

To reduce the risk of thrombosis and ischemic strokes, especially in individuals with hemoglobinopathies like sickle cell anemia. Controlling hypertension is essential for reducing the risk of stroke in anemic patients, as anemia-induced hypoxia may further exacerbate blood pressure dysregulation^[31,32]^.

## Conclusion

The interplay between anemia and cerebrovascular disease presents a complex and multifaceted challenge to both diagnosis and management. The mechanisms through which anemia exacerbates cerebrovascular complications – such as hypoxia, oxidative stress, endothelial dysfunction, and blood-brain barrier disruption – underscore the critical importance of early detection and tailored management strategies. Given the prevalence of both conditions globally and their significant impact on public health, understanding and addressing anemia in the context of cerebrovascular disease is essential for improving patient outcomes. While current management strategies, including correction of anemia and control of cerebrovascular risk factors, have shown promise, there remains a need for further research to elucidate the precise molecular mechanisms linking anemia and cerebrovascular disease. Future studies should focus on exploring the genetic and epigenetic factors that contribute to the development of anemia-related cerebrovascular complications, as well as the long-term effects of anemia on brain health, particularly in high-risk populations. Moreover, advancements in personalized medicine, such as more refined diagnostic tools and targeted therapeutic interventions, hold great potential in improving outcomes for patients with both anemia and cerebrovascular disease.

## Data Availability

Not applicable.
